# Supplementary material for: Anlotinib Exerts Inhibitory Effects against Cisplatin-Resistant Ovarian Cancer In Vitro and In Vivo
Source: Molecules. 2022 Dec 14;27(24):8873. doi: 10.3390/molecules27248873 (PMC9785832; doi:10.3390/molecules27248873)
Supplement: Supplementary file 1 [file molecules-27-08873-s001.zip › molecules-1996641-supplementary.pdf]

Supplementary Materials

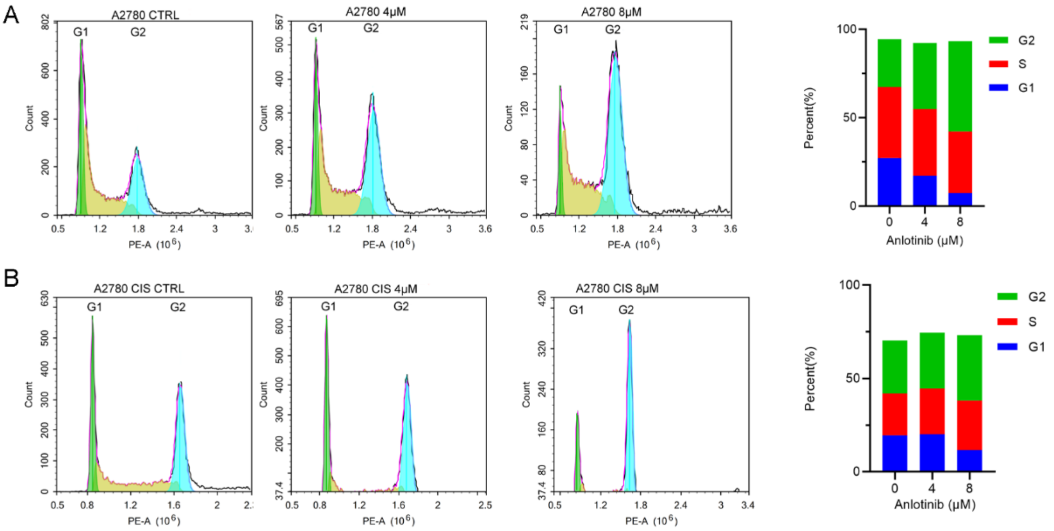

Anlotinib inhibited proliferation by arresting the cell cycle in the G2/M phase.

A2780 cells were treated with anlotinib as indicated concentration. Cell cycle was determined using Flow Cytometry (FCM) analysis.
